# From Diagnosis to Emergency: Mediastinal Abscess After Endobronchial Ultrasound-Guided Transbronchial Needle Aspiration in a Patient With Sarcoidosis

**DOI:** 10.7759/cureus.75584

**Published:** 2024-12-12

**Authors:** Joana Castro Vieira, Mafalda Maria Santos, Mariana Simão de Magalhães, João Vieira Afonso, Ana Cristina Teotónio

**Affiliations:** 1 Internal Medicine, Unidade Local de Saúde do Oeste – Hospital Distrital de Caldas da Rainha, Caldas da Rainha, PRT

**Keywords:** endobronchial ultrasound (ebus), lymphadenopathy, mediastinal abscess, non-caseating granulomas, post-ebus complications, pulmonary inflammatory diseases, sarcoidosis

## Abstract

Sarcoidosis is a systemic granulomatous disease of unknown etiology, primarily affecting the lungs and the lymphatic system. Its diagnosis is challenging, and in many cases, it requires histopathological confirmation through the identification of non-caseating granulomas. The presented case illustrates its diagnostic complexity and highlights a rare, delayed complication associated with endobronchial ultrasound-guided transbronchial needle aspiration (EBUS-TBNA). The patient developed a mediastinal abscess, a serious and uncommon post-procedural event, which likely resulted from microperforation of the bronchial wall during the aspiration. Symptoms developed several days after the procedure, with fever, chest pain, and signs of mediastinal infection. This case emphasizes the need for heightened clinical awareness and careful monitoring following EBUS-TBNA.

## Introduction

Sarcoidosis is a chronic, systemic inflammatory disease characterized by the formation of non-caseating granulomas. It is more prevalent in melanodermic women under 50 years of age. Up to 60% of cases are asymptomatic and are diagnosed incidentally. Respiratory and constitutional symptoms are the most common among symptomatic patients, and extra-pulmonary involvement is frequent, affecting organs such as the eyes, liver, heart, nervous system, and kidneys. Usually, extra-hepatic gastrointestinal involvement is rare. Diagnosis typically requires the exclusion of other conditions, and in many cases, depends on histopathology to confirm the presence of granulomas [1,2].

Due to its variable and nonspecific presentation, sarcoidosis can be mistaken for other diseases, leading to a diagnostic delay. Endobronchial ultrasound-guided transbronchial needle aspiration (EBUS-TBNA) is a minimally invasive technique used for diagnosing lymph nodes and pulmonary tumors. When combined with real-time ultrasound, it improves accuracy, reduces risks, and is generally safer [3].

In this report, we describe a complex case that illustrates the need for a multidisciplinary approach, detailed diagnostic investigation, and the complications resulting from the EBUS-TBNA study.

## Case presentation

**Figure 1:**
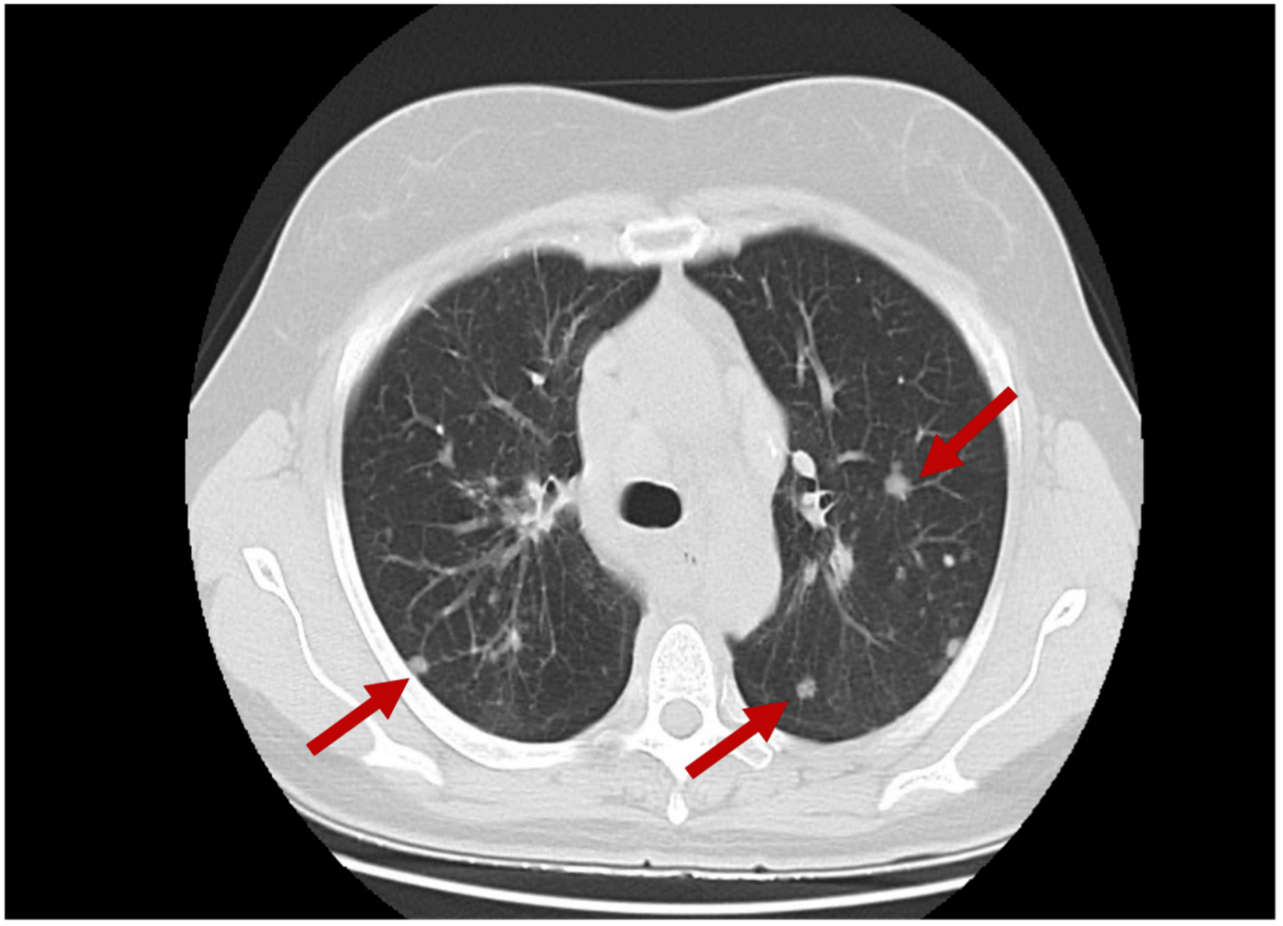
Chest CT (axial view) showing multiple solid and subsolid pulmonary nodules (red arrows).

A 53-year-old female was referred to the Internal Medicine external consult with complaints of fatigue and dyspnea. Chest computed tomography (CT) revealed dispersed solid and subsolid pulmonary nodules in both lungs (Figure 1), associated with lymphadenopathy at the mediastinal and hilar levels. She had no significant medical history and wasn’t on any regular medication.

**Figure 2:**
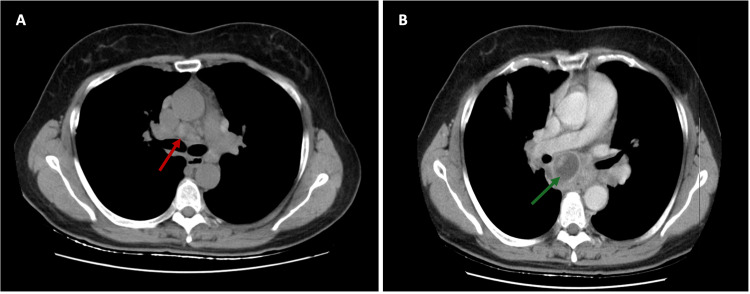
Chest CT images (axial view) showing (A) Subcarinal adenomegaly with 17 mm in its short axis before EBUS-TBNA (red arrow) and (B) Posterior mediastinal abscess after EBUS-TBNA (green arrow). EBUS-TBNA: endobronchial ultrasound-guided transbronchial needle aspiration

Complementary investigations including laboratory tests, serologies, angiotensin-converting enzyme (ACE), interferon-gamma release assay (IGRA), myeloperoxidase (MPO), proteinase 3 (PR3), and antinuclear antibodies (ANA) were carried out, all of which were negative. Transthoracic echocardiogram (TTE) was normal for her age, and a positron emission tomography (PET) scan was performed to exclude a neoplastic etiology. The PET scan showed lymph node activity and bilateral pulmonary parenchymal involvement, suggesting a possible inflammatory disease such as sarcoidosis or a lymphoproliferative disorder. Given the possibility of lymphoproliferative disease and with a subcarinal adenomegaly seen on CT (Figure 2A), an EBUS-TBNA was performed, and lymph nodes from station 7 (subcarinal) and 11L (left interlobar nodes) were sampled. Cytology and pathology reports did not reveal atypical cells or changes suggestive of granulomatous disease.

One month after the EBUS-TBNA, the patient presented with food impaction, retrosternal pain, fever, and elevated inflammatory markers, leading to an emergency room visit. A CT scan revealed a posterior mediastinal abscess (Figure 2B).

Due to the severity of the finding, the case was discussed with the Cardiothoracic Surgery team. The patient underwent drainage of a mediastinal abscess located in the subcarinal region. A necrotic lymph node conglomerate with purulent content was identified and debrided. Biopsies were obtained from station 7 lymph nodes. Additionally, a wedge resection of the right upper lobe (RUL) was performed to remove multiple nodular lesions. Histopathological examination confirmed the diagnosis of sarcoidosis, showing non-necrotizing granulomas in both the pulmonary parenchyma and lymph nodes.

The patient was hospitalized for 14 days and initially started on empirical piperacillin-tazobactam 4.5 g IV every eight hours. Later, *Streptococcus constellatus *was isolated from the purulent material obtained in the subcarinal abscess cavity. The antibiotic therapy was continued as the organism was found to be sensitive to piperacillin-tazobactam. The patient showed subsequent clinical and analytical improvement. Upon discharge, the patient was prescribed oral amoxicillin-clavulanate 875 mg + 125 mg every 12 hours, to complete a two-week course.

After discharge, due to persistent fatigue with medium exertion and changes in pulmonary function tests, the decision was made to initiate treatment with oral prednisolone (20-40 mg/day) with gradual tapering over six to nine months. The patient underwent screening for extrapulmonary sarcoidosis, including liver function tests, blood urea nitrogen, creatinine, glucose, electrolytes, serum calcium, ECG, and TTE, and all were normal. No cutaneous abnormalities were noted, and an ophthalmologic examination revealed no intraocular inflammation.

## Discussion

The incidence of sarcoidosis ranges from 2.3 to 17.8 per 100,000 people annually, depending on the region and patient cohort, making it a rare disease [4]. Its presentation is variable, often mimicking other conditions. It is a multisystem disease characterized by fatigue, memory loss, shortness of breath, cough, pain, and dizziness [5]. Diagnostic tests are often suggestive but not definitive. While the literature highlights difficulties in diagnosing sarcoidosis, prognosis depends on factors such as organ involvement, treatment response, ethnicity, and age [6].

There is no specific test for diagnosing sarcoidosis, requiring criteria such as clinical evidence, imaging, non-caseating granulomas on biopsy, and the exclusion of other conditions. The diagnostic process is complex and time-consuming, leading to delays in diagnosis [7].

In this clinical case, the diagnosis of sarcoidosis was fortuitously reached following a rare complication. EBUS-TBNA is a minimally invasive technique used to diagnose mediastinal and hilar lymph nodes, pulmonary tumors near the airways, and masses at the lung apex close to the trachea [8]. Using real-time ultrasound improves sampling accuracy and reduces the risk of bleeding, being relatively safe and effective. EBUS-TBNA has a low complication rate; however, as reported in this case, complications can occur, including infectious complications such as mediastinitis, as well as pericarditis, hemorrhage, and pneumothorax. The incidence of complications varies depending on the medical environment and the operator [9].

While pneumothorax and pneumomediastinum are typically treated conservatively [10], mediastinitis often requires prolonged hospitalization, antibiotic therapy, and sometimes surgical intervention [11]. Fatalities are rare, occurring in less than 1% of major studies [12,13].

After discharge, the patient began treatment with corticosteroids due to persistent fatigue and changes in pulmonary function tests. Treatment for sarcoidosis is indicated when associated with hypercalcemia, potential cardiac or neurological involvement, or severe symptoms such as organ dysfunction [14]. If no clinical improvement is evident, increasing the dose of prednisolone or initiating immunosuppressive therapy with methotrexate should be considered for disease control and to prevent long-term complications [15].

## Conclusions

This case highlights the diagnostic challenges of sarcoidosis, with a more rapid diagnosis achieved following a rare and potentially fatal complication from EBUS-TBNA. Although this is a minimally invasive procedure associated with a low complication rate, severe complications such as mediastinitis have been reported. Given the growing use of EBUS-TBNA, it is crucial to establish an educational system to ensure its safe and effective performance and maintain vigilance for potential complications.
